# Towards characterization of cell culture conditions for reliable proteomic analysis: in vitro studies on A549, differentiated THP-1, and NR8383 cell lines

**DOI:** 10.1007/s00204-024-03858-4

**Published:** 2024-09-12

**Authors:** Rico Ledwith, Tobias Stobernack, Antje Bergert, Aileen Bahl, Mario Pink, Andrea Haase, Verónica I. Dumit

**Affiliations:** 1 Present Address: Department of Chemical and Product Safety, German Federal Institute for Risk Assessment (BfR), Berlin, Germany; 2 Present Address: Institute of Pharmacy, Freie Universität Berlin, Berlin, Germany

**Keywords:** New approach methodologies (NAMs), Toxicity mechanisms, Cell culture conditions,, THP-1 differentiation process, Passage numbers

## Abstract

Proteomic investigations result in high dimensional datasets, but integration or comparison of different studies is hampered by high variances due to different experimental setups. In addition, cell culture conditions can have a huge impact on the outcome. This study systematically investigates the impact of experimental parameters on the proteomic profiles of commonly used cell lines—A549, differentiated THP-1 macrophage-like cells, and NR8383—for toxicity studies. The work focuses on analyzing the influence at the proteome level of cell culture setup involving different vessels, cell passage numbers, and post-differentiation harvesting time, aiming to improve the reliability of proteomic analyses for hazard assessment. Mass-spectrometry-based proteomics was utilized for accurate protein quantification by means of a label-free approach. Our results showed that significant proteome variations occur when cells are cultivated under different setups. Further analysis of these variations revealed their association to specific cellular pathways related to protein misfolding, oxidative stress, and proteasome activity. Conversely, the influence of cell passage numbers on the proteome is minor, suggesting a reliable range for conducting reproducible biological replicates. Notable, substantial proteome alterations occur over-time post-differentiation of dTHP-1 cells, particularly impacting pathways crucial for macrophage function. This finding is key for the interpretation of experimental results. These results highlight the need for standardized culture conditions in proteomic-based evaluations of treatment effects to ensure reliable results, a prerequisite for achieving regulatory acceptance of proteomics data.

**Supplementary Information:**

The online version contains supplementary material available at 10.1007/s00204-024-03858-4.

## Introduction

The current hazard assessment of substances relies heavily on animal testing, which has limitations, such as long testing periods and ethical concerns (Miccoli et al. 2022; Schmeisser et al. 2023). These limitations can conflict with the fast pace of technological advancement (Doak et al. 2022; Fischer et al. 2020). Consequentially, there has been a growing demand for new approach methodologies (NAMs) to animal testing that can predict substance toxicity reliably (ECHA 2016; Jagiello et al. 2023). A NAM refers to any animal-free methods including in silico, in vitro, or *in chemico* approaches that support chemical risk assessment. Contrary to animal-based experiments, NAMs are capable of delivering quicker results from different read-outs simultaneously, enhancing their efficiency. Additionally, NAMs allow for the integration of various assays including different cell models and enable the bypassing of challenges associated with interspecies variation. This is particularly important in ensuring extrapolation of data from experimental animals to humans. Thus, NAMs are able to keep up with the rapid pace of innovation due to the high-throughput capacity, indispensable for the evaluation and safety assessment of new substances and (nano-)materials.

Over recent decades, significant progress has been made in the development of NAMs with wide application in both basic and applied research, as well as in pre-regulatory contexts such as screening and prioritization (Ruijter et al. 2023). The key to broadening this acceptance lies in improving the standardization and validation of NAMs, which is crucial for ensuring their reliability and relevance across various settings and applications (Schmeisser et al. 2023). Hitherto, the regulatory acceptance of NAMs is still limited, largely confined to the assessment of acute effects, such as skin and eye irritation (Stucki et al. 2022). To assess systemic and chronic endpoints, a deep understanding of toxicity mechanisms is required that allows for the development of an effective battery of NAMs organized into structured frameworks. One key approach employed in this context is the Adverse Outcome Pathway (AOP) concept, which facilitates the design of tailored approaches to address complex toxicological endpoints effectively (Bajard et al. 2023; Halappanavar et al. 2020; OECD 2018). AOPs describe a series of key events that span different biological levels, starting from an initial molecular event to a discernible adverse outcome (Halappanavar et al. 2020).

High-content techniques such as omic methods, have been central for the development of AOPs (Brockmeier et al. 2017; Vinken 2019) due to their ability to identify and quantify cellular changes caused by the substance (Dumit et al. 2024), and therefore, aid in advancing the development of NAMs by shedding light on the mechanisms of substance toxicity. Omic data provide information on genes, proteins, metabolites, and related pathways that can be used to select key biomarkers relevant to toxicity (Brockmeier et al. 2017). Therefore, employing proteomics-based methods offers insights into cellular processes that undergo changes in response to a test substance, which can then be effectively further characterized using NAMs (Amorim et al. 2023; Stobernack et al. 2024). Among the frequently employed omics approaches for hazard characterization, transcriptomics stands out as the most advanced technique. This is due to its streamlined data generation and analysis processes (Canzler et al. 2020), which allow results to be compared across different laboratory contexts. On the other hand, proteomics, though not widely applied due to the complex data analysis required, has the potential to provide a more accurate depiction of the phenotype and cellular processes (Bahl et al. 2023; Canzler et al. 2020). Drastic changes at the transcriptome level do not always translate into detectable alterations at the protein level (Canzler et al. 2020), because proteins are subject to further regulatory mechanisms that can significantly influence their abundance, thereby more accurately reflecting the effects of the evaluated substance. However, it is important to assess the robustness of these measurements in the experimental setups. As with other important measurements, the evaluation of proteomic profiles in toxicity assessments must be done in the context of experimental conditions. A significant research gap and lack of awareness exist in understanding how these conditions influence the proteomic profiles of cell lines, particularly given the variability in laboratory parameters, such as different instrument setups and quantification methods, and measurement conditions, among others, which poses a challenge to the interpretation of proteomic data in toxicological studies.

The human lung epithelial A549 cell line, the human monocytic THP-1 cell line that can be differentiated into macrophage-like cells (dTHP-1), and the rat alveolar macrophage NR8383 cell line are increasingly used as lung models for in vitro studies (Di Ianni et al. 2022; Faber and McCullough 2018). They are particularly important for nanomaterial toxicity research, since inhalation is the most critical route of exposure. Due to their small size, nanoparticles can penetrate deep into the lungs, reaching the alveoli. Therefore, understanding their interaction with and the responses of alveolar epithelial cells and alveolar macrophages is of toxicological interest (Bierkandt et al. 2018). A549 cells are favored for their similarity to type II alveolar epithelial cells, making them a pertinent model for investigating lung-related toxicity. Since human alveolar macrophage cell lines are lacking, dTHP-1 cells are often utilized to study immunological responses of the lung. Additionally, including a rat alveolar macrophage cell line in this study is warranted due to its high predictivity for short-term inhalation toxicity in nanomaterial studies (Wiemann et al. 2016). However, these cell models are employed under variable conditions, which can affect the outcomes of toxicological assays (Aldo et al. 2013; Arodin Selenius et al. 2019; Pérez-Cano et al. 2003). This underscores the importance of understanding how various cell culture conditions impact their proteomic profiles. Such insights would facilitate the interpretation of new findings in the context of existing knowledge.

Our study aims to investigate the influence of different experimental parameters on the proteomic profile of unexposed A549, dTHP-1 and NR8383 cells, thereby raising awareness of how varying cell culture conditions impact the proteome. Our work focused on the effects of cell culture setups, specifically comparing the seeding of cells in 6 well plate (6WP)-wells vs. 10 cm cell culture dishes. These setups were chosen due to their widespread use in generating sufficient cell material for MS-based proteomics in toxicity screenings. Additionally, we examined proteomic changes associated with different cell passage numbers, a critical factor considering that toxicological screenings often require biological replicates from various passage numbers. Furthermore, our study assessed the proteomic variations in dTHP-1 cells at different post-differentiation times. This study considers the various settings commonly used across labs, albeit within a standard range of conditions. Understanding how various conditions specific to cell culture experiments can influence the proteome is crucial for establishing stable and consistent models, which may be part of strategies for deepening our knowledge of cellular toxicity mechanisms. Improving standardization of experimental conditions is expected to increase the regulatory acceptance and implementation of proteomics as part of NAMs.

## Materials and methods

### Cell lines and culture conditions

A549 (ATCC, USA, CRM-CCL-185): the human lung adenocarcinoma epithelial cell line was obtained from the American Type Culture Collection, Virginia, USA. Cells were grown in MEM GlutaMAX (Gibco, USA, 41,090–028) supplemented with 10% fetal calf serum (FCS) GOOD, (PAN Biotech, Germany, P40-37500) and 1% penicillin–streptomycin, (PAN Biotech, Germany, P06-07100).

THP-1 (DSMZ, Germany, ACC 16): the human monocytic leukemia cell line was obtained from the German Collection of Microorganisms and Cell Cultures GmbH, Braunschweig, Germany. Cells were grown in Roswell Park Memorial Institute medium (RPMI 1640), (PAN Biotech, Germany, P04-17500) supplemented with 10% FCS GOOD, 1% penicillin–streptomycin, 1% L-Glutamine (PAN Biotech, Germany, P04-80100)., 10 mM HEPES buffer (PAN Biotech, Germany, P05-01100) and 1 mM sodium pyruvate (PAN Biotech, Germany, P04-43100).

THP-1 differentiation: THP-1 cells were differentiated to macrophages for 48 h with 100 nM of PMA (Sigma Aldrich, Germany, P1585) in RPMI medium. Following this 48-h period, PMA containing medium was removed and the cells was replenished with fresh RPMI medium.

NR8383 (ATCC, USA, AgC11 × 3A, NR8383.1): rat macrophage cell line originally isolated from the lungs of a normal rat, were obtained from the American Type Culture Collection, Virginia, USA. Cells were grown in Ham’s F-12 K (Kaighn’s) Medium (Gibco, USA, 21,127,022), supplemented with 15% FCS GOOD and 1% penicillin–streptomycin.

After thawing from cryopreservation each cell line was kept in cell culture for a total of four passages, to allow for a degree of phenotypical stabilization. Cell lines were then passaged twice per week, every 3 or 4 days. All cell lines and were incubated at 37 °C, 5% CO_2_. Cell cultures underwent routine testing for mycoplasma contamination, consistently yielding negative results throughout the experimental period.

### Cell culture setups

In a first step, we evaluated the effects of culturing cells of three different cell lines in different cell culture vessels, i.e., 6WP-wells and 10 cm dishes. To address discrepancies between the recommended cell numbers and cell culture media (CCM) volumes by cell line providers, we used cell numbers and CCM volumes that align with standard recommendations, which are also commonly utilized in scientific literature. An overview of these conditions is listed in Tables 1 and 2. The cell density was kept constant for both cell culture vessels (Table 1) and is calculated considering cell number divided by vessel’s area. Due to the different CCM volumes used, 6WP-wells had higher volume to surface area ratio (mL/cm^2^) and CCM filling height compared to the 10 cm dishes (Table 2). Thus, these represent key differences of the experimental setup, which were assessed. 24 h after seeding the cells in the corresponding vessels the cells were harvested and processed for proteomic analysis.

**Table 1:** Comparison of the cell seeding densities for the different cell lines in both culture vessels

	6WP-well	10 cm dish	Cell/cm^2^ (both vessels)
A549	2.1 × 10^5^	1.4 × 10^6^	2.32 × 10^4^
dTHP-1	4.5 × 10^5^	3.0 × 10^6^	4.99 × 10^4^
NR8383	1.2 × 10^6^	8.0 × 10^6^	1.33 × 10^5^

**Table 2:** Metrics for each vessel comparing the different parameters

Vessel	Diameter (cm)	Border (cm)	Surface (cm^2^)	Border /surface area ratio	Volume (mL)	Volume/surface area ratio (mL/cm^2^)	Filling height (cm)
10 cm dish	8.75	27.48	60.1	1: 0.45	10	0.166	0.166
6WP-well	3.39	10.65	9.03	1: 1.18	3	0.332	0.332

Cells were seeded as detailed in Table 1, across three biological replicates derived from distinct cell passage numbers. For each biological replicate, we performed three technical replicates, using cells from the same passage number but grown in separate culture vessels. The cell density was maintained constant across different vessels relative to their surface area.

The characteristics of the cell culture vessels used for the experiments are presented in Table 2.

### The cell passage number range

In a second step, we investigated alterations in the proteomes of A549, dTHP-1, and NR8383 cells focusing on variations across different subcultures of the same stock. The cells of different passage number were grown in 10-cm dishes or 6WP-wells and harvested 24 h after cell seeding. The cultivation times were adapted to fit the practical constraints of the laboratory schedule, ensuring that cell splitting could be integrated smoothly into the regular workweek. Specifically, A549 cells for five passages (2.5 weeks), and dTHP-1 cells for seven passages (3.5 weeks) and NR8383 cells were cultured for up to three passages (equivalent to 1.5 weeks). This approach reflects a realistic scenario in toxicological research, where experiments must often be designed to accommodate the standard workweek, thereby ensuring to obtain three biological replicates for robust toxicological testing.

### Differentiation process of THP-1 cells

In the third step, we investigated the proteomic changes occurring in dTHP-1 cells following the completion of the PMA-induced differentiation process. Innately monocytic cells, THP-1 acquire a macrophage-like phenotype and functional state through differentiation induced by PMA, a compound from the phorbol ester family. However, a notable limitation of differentiating THP-1 cells by PMA is that they enter an (pro-)inflammatory state, a change anticipated to be reflected in their proteomic profile. Following the standard procedure for THP-1 cell differentiation (Cam and de Mejia 2012; De et al. 2021), after the 48-h differentiation period with PMA, the PMA-containing medium was removed and the cells were supplemented with PMA-free CCM. Cells were then harvested at three distinct time points: 6, 24 and 48 h post-PMA treatment.

### MS sample preparation, liquid chromatography–electrospray ionization–tandem mass spectrometry (LC–ESI–MS/MS) measurements

Cell pellets were harvested, lysed and digested for proteomics measurements using the iST kit (PreOmics, Germany, P.O.00030). Desalted peptides were reconstituted in 0.1% (v/v) TFA, 5% (v/v) acetonitrile to a final concentration of 50 ng/µl and transferred to vials with glass inserts. LC–MS analyses were performed on an UltiMate 3000 RLSCnano system (Thermo Scientific, USA) connected to an Orbitrap QExactivePlus (Thermo Scientific, USA) mass spectrometer.

The LC system was coupled to the mass spectrometer via a nanospray flex ion source equipped with a stainless-steel emitter (Thermo Scientific, USA). Samples were injected (250 µg) and concentrated on an Acclaim PepMap100 C18 trap column (3 μm, 100 Å, 75 μm i.d. × 2 cm, Thermo Scientific) equilibrated (5 µL/min, 5 min, 45 °C) with 0.05% TFA, 2% acetonitrile in water. After switching the trap column inline, peptides were separated on an Acclaim PepMap100 C18 column (2 μm, 100 Å, 75 μm i.d. × 25 cm, Thermo Scientific) at an eluent flow rate of 0.3 µL/min using a two linear gradient (5 to 35% B in 90 min, 35 to 50% in 5 min). Mobile phase A contained 0.1% formic acid in water, and mobile phase B contained 0.1% formic acid in 80% acetonitrile. Non-targeted analysis was performed in a data-dependent acquisition (DDA) mode, fragmenting the ten most abundant, multiply charged ions, with dynamic exclusion time set to 60 s. Each sample was measured in three analytical replicates. A full list of instrument parameters is given in Supplementary Table 1.

### Protein identification and data analysis

Mass spectrometric data from each LC–MS run were analyzed using the MaxQuant software (Cox and Mann 2008; Tyanova et al. 2016) (Version 1.6.14). The identification of proteins was performed using the MaxQuant-implemented Andromeda search engine against a reference *Homo sapiens* proteome. Precursor and fragment mass tolerance were set to 7 ppm and 0.5 Da, respectively. Variable (methionine oxidation and N-terminal acetylation) and fixed modifications (cysteine carbamidomethylation) were set for the search and trypsin with a maximum of three missed cleavages was chosen for searching. The minimum peptide length was set to seven amino acids and false discovery rate (FDR) for peptide and protein identification was set to 0.01. Proteins were quantified via label-free quantification (LFQ) as detailed in Stobernack et al. 2024 (Stobernack et al. 2024).

### Statistical analysis

**Fig. 1:**
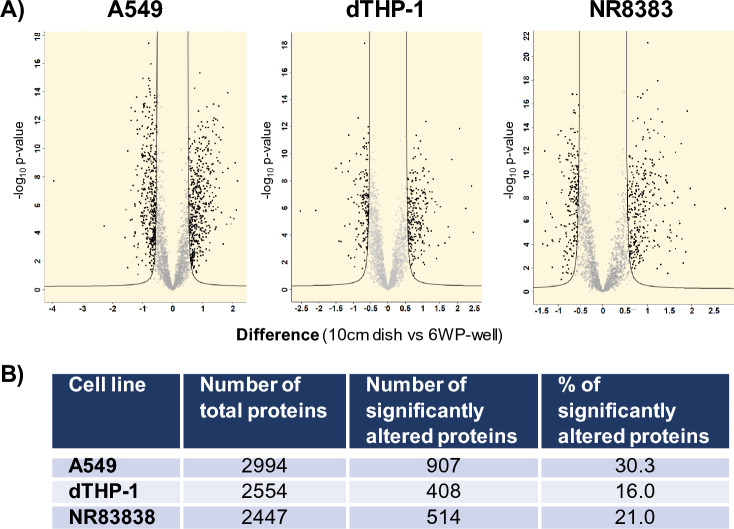
Effect of the cell culture setup at the proteome level assessed 24 h after cell seeding **A** Volcano plots depicting the impact of the cell culture setup selection (10 cm dishes vs. 6WP-wells) on the proteome profiles of the different cell lines: A549, dTHP-1 and NR8383. **B** Total proteins detected per cell line and proteins significantly altered upon seeding in different vessels, presented as both the total count and percentage

All conducted experiments included three biological replicates for each sample and were analyzed in three technical replicates. In this study, protein LFQ intensities were pre-processed by log2 transformation. The Perseus software (Tyanova et al. 2016) was utilized to identify proteins with significant alterations between two compared conditions or groups. We identified significantly altered proteins using FDR settings of 0.01 and s0 set at 0.1, as employed in multi-volcano analysis, based on criteria established by Rudolph et al., 2019 (Rudolph and Cox 2019). The software provides an analysis summary in a data matrix format, which includes t-test significance, -Log t-test values, p-values, and t-test differences. Volcano plots, as shown in Fig. 1, graphically represent the significance and magnitude of changes in protein expression, combining the functions of the two-sample t-test and the scatter plot. Significantly altered proteins were then subjected to KEGG pathway analysis using the R package enrichR (Kuleshov et al. 2016). The AnnotationDBI package was used to convert Uniprot IDs to Entrez IDs. Prism 9.3.1 (GraphPad Software, San Diego, CA, USA) and Excel (Office Professional 2021 Plus, Microsoft, Redmond, USA) were used for calculations, filtering, and graphical display.

## Results

### The cell culture setup extensively affects the proteome

A graphic representation of the results is shown in Fig. 1A). Each volcano plot compares the levels of each detected protein in both cell culture vessel for each one of the cell lines included in the analysis. Individual proteins are visually represented, with its position determined by both its fold change (x-axis) and its statistical significance (y-axis). Notably, threshold curvature lines on the plot denote the specific cutoffs for significance, as determined by the false discovery rate (FDR) = 1% and *s*_0_ = 0.1 values. Supplementary Table 2 includes the list of all identified proteins, highlighting those that are significantly altered for each cell line. Fig. 1B) provides a comprehensive summary of the detected protein counts per cell line, including the counts of proteins exhibiting statistically significant changes, meeting the same cutoff criteria outlined above.

Among the evaluated cell lines, A549 exhibited the highest percentage of variability, since it showed the highest number of significantly altered proteins, followed by NR8383 and dTHP-1. This offers a tentative ranking of cell line susceptibility to the cell culture setup. To understand the biological functions and interactions of the significantly altered proteins we performed a KEGG pathway (Kanehisa and Goto 2000) analysis. This approach provides insights into the underlying mechanisms of cellular processes being affected by the different cell culture setups in each cell line.

Table 3 enumerates the KEGG pathways altered by different cell culture setups across the evaluated cell lines. Supplementary Table 3 includes further KEGG pathways that showed to be regulated in only two or one of the evaluated cell lines. Notably, six pathways are shared among all three tested cell lines, with four of these pathways reflecting proteomic changes similar to those seen in neurodegenerative disorders. The implicated proteins are not exclusively related to a single disease but are involved in a range of biological functions, including cellular stress responses, mechanisms of protein folding and degradation. Among these shared pathways, the involvement of the proteasome pathway is particularly noteworthy due to its essential role in protein degradation and turnover within the cell. The consistency of proteome alterations across the three distinct cell lines underscores the significant impact of cell culture conditions on the proteomic landscape.

**Table 3:** Altered KEGG pathways affected by the cell culture setup in evaluated cell lines. Numbers represent FDRs of the corresponding pathway if regulated for that particular cell line

KEGG pathway	A549	dTHP-1	NR8383
Parkinson disease	2,45E-08	1,77E-02	1,54E-10
Amyotrophic lateral sclerosis	7,40E-09	3,98E-02	1,54E-10
Prion disease	8,93E-08	1,77E-02	6,48E-09
Pathways of neurodegeneration—multiple diseases	1,49E-05	3,98E-02	6,69E-08
Proteasome	2,30E-07	1,40E-02	4,64E-05
Salmonella infection	3,17E-02	3,98E-02	4,12E-02

### The cell passage number range had no notable variances on protein levels

The investigated cell lines displayed notable proteomic stability within the study period, as highlighted in Table 4, which provides an overview of the amount of significantly changed proteins. Among the cell lines, A549 exhibited the highest number of significant protein alterations, particularly in 6WP-wells compared to 10 cm dishes. In contrast, the NR8383 cell line showed negligible differences across cell passage numbers for the two types of culture vessels. Despite having the broadest range of passage numbers evaluated, the dTHP-1 cell model revealed no notable variances in protein levels.

**Table 4:** Effect of the cell passage number. Number of significantly altered proteins for A549, dTHP-1 and NR8383 cell lines when comparing different cell passage numbers, both when cultured in 10 cm dishes and 6WP-wells. The significance criteria used were FDR = 0.01 and *s*_0_ = 0.1. Numerical values within the cells indicate the total count of proteins showing significant alterations, and numbers in parentheses correspond to the percentage of significantly altered proteins relative to the total number of detected proteins (for A549 = 2994, for dTHP-1 = 2554 and for NR8383 = 2447) in each specific cell line and cell passage comparison

Cell Line	Passage number	10 cm dish	6-WP well
A549	First **(day 0)** vs. Fifth **(+ 17 days)**	29 (1.0%)	269 (9.0%)
dTHP-1	First vs. Seventh **(+ 24 days)**	80 (3.1%)	107 (4.2%)
NR8383	First vs. Third **(+ 10 days)**	5 (0.2%)	52 (2.1%)

### Strong proteomic changes in dTHP-1 cells happen during the differentiation process

To study the proteomic changes occurring in dTHP-1 cells following the completion of the PMA-induced differentiation process, we analyzed the dTHP-1 cell proteome at various times of the differentiation, following the PMA removal. Understanding the proteome dynamics of the cells during the experimental time-lapse is crucial for interpreting experiments evaluating treatments at different time-points.

In total, 3255 proteins were detected by MS-based proteomics. Table 5 illustrates the total number and percentage of proteins that are significantly altered when comparing the proteomes of the dTHP-1 cells harvested at different time points post-PMA treatment, in both 10 cm dishes and 6WP-wells.

The data depicted in the Table 5 highlights the significant role of experimental conditions on the proteomic profile changes in dTHP-1 cells. A striking 21.2% of the total of 3255 detected proteins were altered when comparing 6-h and 48-h post-PMA treatment periods in 6-WP wells, suggesting a dynamic proteomic response over time. However, even more pronounced changes are evident in the 10-cm dish format, with more than 30% of the protein levels being affected. This indicates that the cell culture environment substantially influences the extent of proteomic alterations.

**Table 5:** Proteome changes in dTHP-1 occurring at different post-PMA treatment times. Number of significantly altered proteins when comparing proteome of dTHP-1 cells harvested at different times post-PMA removal, i.e., 6 vs. 48 h, both when cultured in 10 cm dishes and 6-WPs. The significance criteria used was FDR = 0.01 and* s*_0_ = 0.1. Numerical values within the cells represent the total count of proteins showing significant alterations, and figures in parentheses correspond to percentage of significantly altered proteins relative to the total number of detected proteins (3255 in this experiment)

Post-PMA removal time (6 vs. 48 h)	10-cm dish	6-WP well
	1002 (30.8%)	690 (21.2%)

Table 6 shows that irrespective of the cell culture setup, considering a 6WP-well or 10-cm dish, enrichment of KEGG pathways consistently revealed that the same set of pathways undergo dynamic alterations during the post-differentiation time. An extended list of enriched KEGG pathways is included in Supplementary Table 4. Significantly, proteins associated to pathways including “Lysosome,” “Endocytosis,” and “Phagosome” consistently demonstrate alterations happening at different post-differentiation times, as evident by the comparison of 6 vs. 48 h after completion of the differentiation process. This observation suggests that these critical signaling pathways for macrophage function undergo dynamic changes in dTHP-1. This finding holds significance for interpreting experimental results derived from toxicological testing involving dTHP-1 cells treated at various time-points.

**Table 6:** Altered KEGG Pathways observed when dTHP-1 cells harvested at different post-differentiation time points (6 vs. 48 h) in the different cell culture setups were compared

KEGG pathway	6WP-well	10 cm dish
Carbon metabolism	3,96E-05	3,27E-02
Amyotrophic lateral sclerosis	1,34E-04	4,26E-08
Lysosome*	**3,46E-04**	**2,78E-06**
Nucleocytoplasmic transport	3,46E-04	2,78E-06
Endocytosis*	**3,87E-04**	**1,74E-02**
Phagosome*	**1,56E-03**	**1,73E-03**
Diabetic cardiomyopathy	2,12E-03	1,04E-02
Prion disease	2,53E-03	3,50E-05
Oxidative phosphorylation	2,53E-03	4,57E-03
Pathways of neurodegeneration—multiple diseases	2,77E-02	2,20E-03
Huntington disease	4,07E-02	5,62E-06
Alzheimer disease	4,42E-02	1,06E-02
Valine, leucine and isoleucine degradation	4,07E-02	2,29E-02

Pathways highlighted in bold and marked with an asterisk (*) are crucial for macrophage function

## Discussion

In this study, we investigated the robustness of cell lines commonly employed as cell models for the lung. Hence, they are often applied for assessing substance toxicity in the context of inhalation (Bessa et al. 2023; Di Ianni et al. 2022; Dumit et al. 2024). Through a series of systematic experiments, we investigated the proteomes of A549, dTHP-1 and NR8383 cells, and how they are influenced by experimental parameters, including the cell culture setup, cell passage numbers, and the harvesting time of dTHP-1 cells post-differentiation. By comparing two conditions or groups, significantly altered proteins can be identified. Focusing on a single protein might be insufficient, yet the relation among different altered proteins can elucidate changes in cellular pathways. This approach, known as pathway enrichment analysis, allows for a more comprehensive understanding of the underlying biological processes that respond to the evaluated conditions. In this work, enrichment of KEGG pathways was performed to show the importance of carefully considering the impact of different cell culture conditions in the experimental design and data interpretation.

### Proteome effects of different cell culture setups

To investigate how the cell culture setups affects a cells proteome, cells from the three distinct cell lines were cultured in 10 cm dishes and 6WP-wells. To obtain comparable data, the cell density was kept constant for both cell culture vessels, thus the number of seeded cells was adjusted according to the growth area of the vessel (Table 1). The used cell culture setups, including the applied volume of CCM used for the different vessels, is summarized in Table 2. These parameters follow those typically recommended for each cell culture setup. This approach results in varying CCM heights in each vessel type. The variation in CCM height could, therefore, contribute to the observed differences in cellular responses and protein expression between the different culture vessels. However, research specifically investigating the impact of CCM heights in different cell culture vessels is limited.

Proteomics analysis on the collected cells revealed that the proteome variations ranged between 16.0% and 30.3% of significantly altered proteins for the evaluated cell lines (Table 3), with A549 displaying the highest percentage. This observation suggests that the cell culture setup can affect the levels of an extensive number of proteins. Interestingly, pathway enrichment analysis among the significantly altered proteins showed a similar response in A549, dTHP-1 and NR8383. Pathways affected in all three cell lines by the cell culture conditions are related to protein misfolding (like aggregation and degradation), oxidative stress, or proteasome activity. This underscores the importance of considering the impact of culture conditions in experimental design and interpreting results in lung toxicity studies.

It has been proposed that cell culture induces oxidative stress on cells, suggesting that cells in culture may activate ROS-dependent signal transduction pathways in a different way than in vivo (Halliwell 2003). Considering our findings, it is possible that varying vessel shapes could influence the degree of oxidative stress experienced by cells, thereby impacting protein misfolding and aggregation across different cell culture setups. It is worth noting that the differences in the CCM height in the different vessels may influence gas exchange, affecting the oxygen diffusion rate (Al-Ani et al. 2018). This variation can potentially lead to oxidative stress conditions, impacting protein oxidation and misfolding, as indicated by the affected KEGG pathways. Although pH can affect protein structure and function, potentially contributing to stress responses and protein misfolding, we do not believe these differences originate from pH variations, as the pH buffering capacity of the CCM volumes used is not a limiting factor under the evaluated conditions over 24 h. Similarly, differences in CCM volume can alter the concentration of nutrients and the accumulation of metabolic waste products, affecting cellular metabolism and stress responses. However, the CCM volume is sufficient to prevent these issues within the evaluated 24-h timeframe.

While these experiments do not definitively determine the most suitable vessel for producing reliable results, our observations regarding protein alterations across different passage numbers suggest that 10 cm dishes yield more consistent results over time. This conclusion is logical, as the larger surface area of a 10 cm plate minimizes the influence of the border, potentially leading to more uniform cell densities. Hence, we suggest utilizing larger vessels to enhance the reproducibility of results.

Regarding which cell line might provide more reliable results, it appears that the proteome of A549 cells is more susceptible to the culture vessel and associated cell culture setup. A549 cells, being a cancer cell line derived from lung carcinoma, may inherently exhibit more variability due to their genetic instability and higher proliferation rates compared to differentiated cell lines like dTHP-1 (which is also a cancer cell model but does not proliferate after differentiation) and NR8383 (not a cancer cell line). Our study aimed at increasing the reliability of toxicological assays using these cell lines. However, determining their relevance requires further investigation, since each one offers different utilities for specific research questions: A549 cells are lung epithelial cells, differentiated THP-1 cells are macrophage-like, and NR8383 cells are alveolar macrophages. As the differentiation process of THP-1 cells has a significant impact on the proteome, as shown in this work, this would suggest that NR8383 cells might be more suitable for investigating immunological processes. However, it is important to note that NR8383 is a rat cell model, whereas dTHP-1 is a human model. In this way, the choice of cell line should be guided by the specific research context and the biological relevance of the cell type. Overall, our work emphasizes the importance of being aware of the variabilities in interpreting results, rather than advocating for a single most robust cell line.

### Proteome changes in cell of different passage number

Additionally, we investigated the influence of passage number on the proteome. Passage number refers to the number of times cells have been sub-cultured. Contrary to the effect caused by the cell culture setup, only negligible proteome alterations were detected among the evaluated passage numbers (Table 4). Of the three assessed cell lines, only A549 exhibited minor differences in protein levels in the evaluated cell passage range. Overall, our results indicate that the examined cell lines show a cell passage number range within which biological replicates can be conducted in a reproducible manner, ensuring to obtain sufficient biological replicates for a robust experimental outcome. Nevertheless, we observed a few notable changes, and therefore, we recommend that specifically for omics studies, it might be advisable to obtain the different biological replicates within a relatively narrow and consistent passage number range to minimize potential variability.

### Proteome changes following post-differentiation process on dTHP-1 cells

The intended differentiation of THP-1 monocytes into macrophage-like cells, typically induced by PMA treatment, is known to initiate significant remodeling effects. However, experiments involving diverse treatments on dTHP-1 cells commonly start upon their differentiation. To investigate the proteomic alterations in dTHP-1 cells post-PMA-induced differentiation, we analyzed their proteome at several time points after PMA removal. Recognizing the dynamic nature of the proteome throughout the experimental duration is critical, particularly for studies evaluating treatment effects at different time-points.

Contrary to the effect of the passage number, the proteome of dTHP-1 cells harvested at different post-PMA removal times, particularly 6 h vs. 48 h, exhibited substantial alterations. This observation underscores the significant dynamic alterations in dTHP-1 cells occurring after the PMA has been removed, suggesting a further ongoing differentiation process. These changes affect as much as 30.8% of the total identified proteins when dTHP-1 cells are cultured in 10-cm dishes, and up to 21.2%, when cell are grown in 6WP-wells (Table 5). The fact that cells cultured in 6WP-wells displayed fewer proteome variations might be related to the volume to surface ratio, specifically the filling height (Table 2). In plates with an increased filling height, the cells may be less affected by the accumulation of metabolic waste products which may become important at 48 h of cell culture. This is particularly notable under a stressful condition as the PMA treatment. Additionally, our results are in agreement to previous finding on the strong effect of varying PMA treatment conditions for the differentiation of THP-1 cells, indicating substantial impact in immune and inflammatory responses (Pinto et al. 2021). Notably, pathways enriched among significantly altered proteins relate to lysosome, endocytosis and phagosome pathways, independently of the cell culture setup.

Our results are important due to the key role of lysosomes in macrophages: Macrophages play a crucial role in engulfing and clearing pathogens, debris, and foreign particles. Given that dTHP-1 cells represent a traditional and valuable in vitro model, particularly due to the scarcity of human macrophage cell lines, it becomes crucial to acknowledge the extent of proteomic changes when employing these cells in studies where comparative analysis of results is critical. Currently, a universally accepted protocol for differentiating or polarizing THP-1 cells into macrophages does not exist (Yasin et al. 2022). Various methodologies incorporate a post-differentiation resting period, ranging from 24 to 120 h (Yasin et al. 2022), which could significantly affect the consistency and reproducibility of research involving differentiated dTHP-1 cells, as indicated by our findings.

In the light of our results, we can conclude that precise timing in sample harvesting and evaluating multiple time points are key to obtaining reliable results, while the specific conditions should be tailored to the objectives of each study. Our results emphasize the need for standardization in experimental design, particularly for the purpose of regulatory frameworks. When planning proteomic experiments, it should be noted that variations in cell culture setups and harvesting times of dTHP-1 cells can contribute to the heterogeneity in protein profiles. It is crucial to account for these factors as potential confounders during data analysis and interpretation, especially when comparing proteomic datasets generated from different experiments or laboratories.

## Conclusion

In conclusion, our study systematically investigated the impact of various experimental parameters on the proteomic profiles of A549, dTHP-1, and NR8383 cell lines, which are commonly employed lung cell models to investigate toxicity in the context of inhalation, like nanomaterial toxicity. Initially, we observed substantial variations in the proteome due to cell culture setup, including vessel type and CCM height, which may influence gas exchange. A549 cells displayed the highest sensitivity to these conditions. Pathway enrichment analysis revealed common responses across the cell lines, particularly in pathways related to oxidative stress and protein misfolding, highlighting the need to consider these variables in experimental design and result interpretation, in particular when investigating oxidative stress-related effects, as it is often the case for nanomaterials. Second, the influence of cell passage numbers on the proteome was relatively minor, suggesting a stable range within which biological replicates can be conducted reliably for robust experimental outcomes. Proteome variations across different cell passages were consistently lower for all evaluated cell lines seeded in 10 cm dishes relative to 6WP-wells. Nevertheless, careful selection of passage numbers is advisable for omics studies to minimize potential variability. However, a notable observation was the substantial proteome dynamic alterations occurring in dTHP-1 cells following the differentiation process, which typically marks the initiation of the experimental phase to evaluate treatment effect. We found that dTHP-1 cells harvested 6 and 48 h after PMA-induced differentiation, exhibited significant alterations in 21.2–30.8% of the overall detected proteins. The extent of protein alterations varied with the type of cell culture vessel used, with a higher volume to surface ratio resulting in lower proteome variation. The protein changes are associated with vesicular trafficking, like lysosome, endocytosis, and phagosome pathways, as seen by enrichment analysis. Given the key role of lysosomes in macrophages, these observations emphasize the need for awareness when employing dTHP-1 cells for toxicological studies. This finding emphasizes the dynamic and nature of the proteome during the differentiation process and the necessity for precise timing in sample harvesting to ensure reproducible results. In summary, our study highlights the strong influence of general cell culture factors on the proteome of commonly used cell lines for inhalation toxicity assessment and emphasizes the importance of methodological standardization to ensure the reliability and reproducibility of results. Addressing these factors is crucial to guarantee consistent outcomes, essential for gaining regulatory acceptance of proteomics data.

## Supplementary Information

Below is the link to the electronic supplementary material.

Supplementary file1 (DOCX 14 KB)

Supplementary file2 (XLSX 12 KB)

Supplementary file3 (XLSX 6573 KB)

Supplementary file4 (XLSX 12 KB)

## Data Availability

Raw proteomic data and the full analysis are available upon request.

## Abbreviations

6WP: 6-Well plate
AOP: Adverse outcome pathway
CCM: Cell culture media
dTHP-1: Differentiated THP-1 macrophage-like cells
ESI: Electrospray ionization
FDR: False discovery rate
KEGG: Kyoto Encyclopedia of Genes and Genomes
LC: Liquid chromatography
MS: Mass spectrometry
NAM: New approach methodology
PMA: Phorbol-12-myristate-13-acetate
