# Supplementary material for: Towards characterization of cell culture conditions for reliable proteomic analysis: in vitro studies on A549, differentiated THP-1, and NR8383 cell lines
Source: Arch Toxicol. 2024 Sep 12;98(12):4021–31. doi: 10.1007/s00204-024-03858-4 (PMC11496344; doi:10.1007/s00204-024-03858-4)
Supplement: Supplementary file 1 — Supplementary file1 (DOCX 14 KB) [file 204_2024_3858_MOESM1_ESM.docx]

**Supplementary Material**

**Towards characterization of cell culture conditions for reliable proteomic analysis: *in vitro* studies on A549, differentiated THP-1, and NR8383 cell lines**

**Rico Ledwith^1^, Tobias Stobernack^1^, Antje Bergert^1^, Aileen Bahl^1^, Mario Pink^1^, Andrea Haase^1^, Verónica I. Dumit^1^***

**^1^**German Federal Institute for Risk Assessment (BfR), Department of Chemical and Product Safety, Berlin, Germany

*Corresponding author: [Veronica.Dumit@bfr.bund.de](file:///\\\\MASNWDATA\\GROUP\\GROUP\\Abteilung-7\\VERTRAULICH\\Nano-Projects\\HARMLESS\\7.%20Paper\\2024\\Methodology\\Veronica.Dumit@bfr.bund.de)

**Supplementary Information**

**Supplementary Table 1:** LC-MS parameters

**Supplementary Table 2:** Identified proteins in A549, dTHP-1 and NR8383 cells cultured in different vessels

**Supplementary Table 3:** Altered KEGG pathways affected by the cell culture setup in evaluated cell lines. Numbers represent FDRs of the corresponding pathway if regulated for that particular cell line

**Supplementary Table 4:** Altered KEGG Pathways observed when comparing dTHP-1 cells harvested at different post-differentiation timepoints (6h vs 48 h) in the different cell culture setups
